# Amphipathic Peptides Impede Lipid Domain Fusion in Phase-Separated Membranes

**DOI:** 10.3390/membranes11110797

**Published:** 2021-10-20

**Authors:** Konstantin V. Pinigin, Timur R. Galimzyanov, Sergey A. Akimov

**Affiliations:** Frumkin Institute of Physical Chemistry and Electrochemistry, Russian Academy of Sciences, 31/4 Leninskiy Prospekt, 119071 Moscow, Russia; gal_timur@yahoo.com

**Keywords:** lipid membrane, theory of elasticity, liquid-ordered domain, domain interaction, amphipathic peptide

## Abstract

Cell membranes are heterogeneous in lipid composition which leads to the phase separation with the formation of nanoscopic liquid-ordered domains, also called rafts. There are multiple cell processes whereby the clustering of these domains into a larger one might be involved, which is responsible for such important processes as signal transduction, polarized sorting, or immune response. Currently, antimicrobial amphipathic peptides are considered promising antimicrobial, antiviral, and anticancer therapeutic agents. Here, within the framework of the classical theory of elasticity adapted for lipid membranes, we investigate how the presence of the peptides in a phase-separated membrane influences the fusion of the domains. We show that the peptides tend to occupy the boundaries of liquid-ordered domains and significantly increase the energy barrier of the domain-domain fusion, which might lead to misregulation of raft clustering and adverse consequences for normal cell processes.

## 1. Introduction

Cell plasma membranes comprise about one hundred types of different lipids [1] and display lateral inhomogeneity resulting from in-plane phase separation [2,3,4,5,6,7,8,9]. Besides, there is much evidence of a liquid-ordered (L_o_)/liquid-disordered (L_d_) phase separation in model lipid membranes, the lipid composition of which resembles the composition of cell plasma membranes [10,11,12,13,14]. The domains of the L_o_ phase are also often called “rafts”, as they resemble a floating platform. A lot of functional roles of the L_o_ domains have been proposed [15,16,17]. From experiments, it is known that various membrane proteins have a different propensity for a particular membrane phase [18] and that this partitioning of the proteins is important for their proper functioning [19,20].

While in model membranes the macroscopic phase separation is frequently observed, domains in cell membranes are nanoscopic [3,5,6]. Misregulation of fusion and fission events of such nanoscopic domains might have adverse consequences for normal cell processes. For example, the malfunctioning of cholesterol biosynthesis as it occurs in Smith-Lemli-Opitz syndrome [21], whereby cholesterol is substituted by its metabolic precursor 7-dehydrocholesterol [22], is accompanied by the increase in the energy barrier for the fusion of L_o_ domains [23]. Although difficult to directly observe experimentally, raft clustering, i.e., the fusion of several nanoscale rafts to a larger one, is proposed to be important in such processes as signal transduction [16,24,25], polarized sorting [26], and creation of immunological synapses [27]. Experiments also show that the raft-raft fusion is involved in neuronal polarity determination [28] and assembly of integrin signaling complexes [29].

Currently, much attention is devoted to antimicrobial peptides as promising antibacterial, antiviral, and anticancer therapeutic agents [30,31,32,33,34,35]. These molecules usually have a positive electric charge, which promotes their selectivity towards negatively charged outer monolayers of bacterial plasma membranes. The peptides create pores and disrupt the membranes leading to cell lysis. Amphipathic antimicrobial peptides also have hydrophobic moiety, which may target them to electrically neutral membranes of eukaryotic cells. That is why the peptides are usually tested for hemolytic activity, which is a macroscopically visible phenomenon. However, even if hemolysis does not occur, the peptides might adversely affect the lateral organization of lipid membranes, which is much harder to detect.

Previously, within the framework of the classical theory of elasticity adapted for lipid membranes, we showed that amphipathic peptides, adsorbed on a lipid bilayer with pre-existing phase separation, tend to accumulate at the boundaries of L_o_ domains [36]. The bilayer of L_o_ domains has a larger hydrophobic thickness than that of the L_d_ surrounding membrane [37,38,39]. Elastic deformations arise at the boundary of L_o_ domains to smooth the step in the bilayer thickness [40,41,42]. This compensation of the hydrophobic mismatch is accompanied by the appearance of regions of a non-zero local curvature even though the membrane surface remains macroscopically flat overall [36]. Amphipathic peptides generate a non-zero local curvature and thus should prefer the regions of the membrane, the local curvature of which best fits the curvature induced by the peptides; such regions naturally occur at the L_o_/L_d_ phase boundary. This drives the adsorbed amphipathic peptides to the boundary of L_o_ domains. Besides, the effective local curvature generated by the peptides leads to the membrane-mediated pairwise repulsion between them in a laterally homogeneous membrane [43,44,45]. Therefore, the presence of amphipathic peptides at the boundaries of L_o_ domains can influence the energy barrier of the domain fusion. If two domains are laterally far from each other, the elastic deformations at their edges are independent, and their energy is additive. However, at a small distance between the domains, these deformations overlap, leading to the membrane-mediated interaction between the domains [23,40,46]. In this work, we explore how the presence of amphipathic peptides at the boundaries of L_o_ domains affects the energy barrier of the domain-domain fusion. Given that amphipathic peptides tend to repel each other and accumulate at the domains’ boundaries, we can qualitatively predict that the fusion barrier should increase in the presence of the peptides, impeding the domain-domain fusion. Quantitative calculations do confirm this prediction.

## 2. Materials and Methods

We aim to explore how amphipathic peptides, embedded into the membrane with the L_d_/L_o_ phase separation, influence the lateral membrane-mediated interaction between L_o_ domains.

We consider each monolayer of a lipid bilayer as a continuum three-dimensional elastic medium. To parameterize the deformations, we introduce the field of unit vectors n, called directors, which characterizes the average orientation of lipid molecules; the vectors are directed from the hydrophilic to the hydrophobic part of the monolayer. This vector field is defined on a special surface, called the neutral surface, where the deformations of bending and stretching are energetically decoupled. This surface lies inside each lipid monolayer of the membrane. As we are interested in local deformations, the lateral extent of which is comparable with the thickness of the monolayer, we account for the deviation of directors from the local normal to the neutral surface, N, which gives rise to the tilt deformation mode, characterized by the tilt vector $T=\frac{n}{Nn}-N$. As lipid molecules can freely diffuse, the lateral shear modulus is set to zero. We also take into account the deformation mode of the lateral stretching-compression, characterized by the relative change of the area per lipid molecule, *α*. Applying the classical elastic energy functional [47] to the lipid monolayer and integrating it over the thickness of the monolayer, we obtain the following expression for the quadratic elastic energy density (per unit area of monolayer neutral surface) [45]:

(1) $$\begin{array}{l} w=\frac{1}{2}k_{m}{\left(\nabla \cdot n-J_{0}\right)}^{2}-\frac{1}{2}k_{m}J_{0}^{2}\\ +\frac{1}{2}k_{t}T_{}^{2}+k_{c}^{}T\cdot (\nabla \nabla \cdot n)+\frac{k_{gr}}{2}{\left(\nabla \nabla \cdot n\right)}^{2}\\ +\frac{1}{2}k_{A}^{}{\left(\alpha -\alpha_{0}\right)}^{2}-\frac{1}{2}k_{A}^{}{\alpha_{0}}^{2}-k_{c}^{}{\left(\nabla \alpha \right)}^{2}\\ +BT\cdot \nabla \alpha +C\nabla \alpha \cdot (\nabla \nabla \cdot n)+\sigma d(\Delta S). \end{array}$$

where, $\nabla \equiv e^{i}\nabla_{i}$ is the surface gradient operator, where $e^{i}$ is the local contravariant basis and $\nabla_{i}$ is the covariant derivative; $J_{0}$ and $\alpha_{0}$ are spontaneous curvature and spontaneous stretching, respectively; B, $k_{t}$, $k_{A}^{}$, $k_{gr}$ are the moduli of bending, tilt, stretching and curvature gradient, respectively; $k_{c}^{}$, A, B, C are the moduli of the corresponding coupling terms between T, ∇∇⋅n, and ∇α; the last term σd(ΔS) accounts for the lateral tension applied to the membrane. This energy functional is an upgraded version of the Hamm and Kozlov’s one [48] that additionally takes into account the second-order term of the tilt-curvature coupling T⋅(∇∇⋅n), initially introduced by Terzi and Deserno in Ref. [49]. Besides, we include other second-order energy contributions of stretching and curvature gradient to make the energy functional energetically stable [45].

### 2.1. Elastic Moduli

To obtain quantitative results, we need to specify the values of the elastic moduli. All energy and length values are set in units of *k*_B_*T* (*T* = 300 K) and nanometers, respectively. We use the values of 1.3 nm and 1.8 nm for the hydrophobic thicknesses of the L_d_ and L_o_ monolayers, respectively [37,39]. For the bending moduli, we use 20 *k*_B_*T* and 10 *k*_B_*T* for the monolayers of the L_o_ and L_d_ phase, respectively [50,51,52,53]; spontaneous curvatures are assumed to be zero in both phases; for the tilt modulus, we use the theoretically estimated value of 12 *k*_B_*T*/nm^2^ [48] for both phases because this modulus should not depend on the type of the phase [48]; for the stretching modulus, we use the value of 30 *k*_B_*T*/nm^2^ [53] for both phases; for the rest of the moduli, we use theoretically estimated values [45] that depend on the hydrophobic thickness h of the considered monolayer: $k_{c}=-\frac{k_{t}h^{2}}{6}$ = −3.4 *k*_B_*T* and 6.5 *k*_B_*T* for the L_d_ and L_o_ monolayers, respectively; $k_{gr}=\frac{k_{t}h^{4}}{20}$ ≈ 1.7 *k*_B_*T*∙nm^2^ and 6.3 *k*_B_*T*∙nm^2^ for the L_d_ and L_o_ monolayers, respectively; $B=-\frac{k_{t}h}{2}$ = −7.8 *k*_B_*T*/nm and −10.8 *k*_B_*T*/nm for the L_d_ and L_o_ monolayers, respectively; $C=\frac{k_{t}h^{3}}{8}$ ≈ 3.3 *k*_B_*T*∙nm and 8.7 *k*_B_*T*∙nm for the L_d_ and L_o_ monolayers, respectively. For the lateral tension σ, we use the value of 0.025 *k*_B_*T*/nm^2^ [54] for both phases; the spontaneous stretching is the ratio of the lateral tension to the stretching modulus, i.e., *α*_0_~8×10^−4^ in both phases.

### 2.2. Parameterization of the System

The elastic deformations induced by membrane inclusions or the hydrophobic thickness mismatch do not exceed several nanometers [36,40,45]. This implies that it is permissible to apply the one-dimensional approach for the description of the membrane-mediated interaction of large enough L_o_ domains, e.g., several tens of nanometers in diameter. In the one-dimensional description, the boundary of the lipid domain is assumed to be an infinite straight line and the elastic energy is given per unit length along the domain boundary. We note, however, that even for smaller L_o_ domains the same description is valid, provided that the appropriate effective length of the interaction is accurately chosen [44].

To parameterize the elastic membrane deformations, we introduce a Cartesian coordinate system *xyz*, the *z*-axis of which is directed perpendicular to the lipid bilayer plane. As we are using the one-dimensional description, the boundary of the L_o_ domain is a straight line, and we direct the *x*-axis and *y*-axis perpendicular to and along this line, respectively. Due to the translational symmetry of the system along the *y*-axis, all deformations depend only on the *x*-coordinate, and the surface gradient operator can be replaced, within the linear approximation, by $\frac{d}{dx}$. To denote the upper and lower monolayers, we use the indices “*u*” and “*l*”, respectively. We express the tilt deformation mode, which by definition is $T=\frac{n}{N\cdot n}-N$, as $T_{u}=n_{u}-\frac{d}{dx}H_{u}$ and $T_{l}=n_{l}+\frac{d}{dx}H_{l}$, where *H* denotes the shape of the neutral surface (the distance from the *xy*-plane to the neutral surface measured along the normal to the *xy*-plane). As lipid monolayers are volumetrically incompressible [55,56,57], we have the following equations for the stretching α [45]: $\alpha_{u}=-\frac{h_{u}}{2}\frac{d}{dx}n_{u}-\frac{H_{u}}{h_{u}}+\frac{M}{h_{u}}+1$ and $\alpha_{b}=-\frac{h_{b}}{2}\frac{d}{dx}n_{b}+\frac{H_{b}}{h_{b}}-\frac{M}{h_{b}}+1$, where M is the shape of the membrane mid-surface. The tension contribution to the elastic energy can be expressed, up to the quadratic order, as $d(\Delta S)\approx \frac{1}{2}{\left(\frac{d}{dx}H\right)}^{2}$ for both monolayers. Using these expressions for the tilt field, stretching, and surface area change, we sum the energies of the upper and lower monolayers and obtain the energy of the bilayer in terms of five functions: $n_{u}$, $n_{b}$, $H_{u}$, $H_{b}$, and M (per unit length along the axis of the translational symmetry):

(2) $$\begin{array}{l} w=\frac{k_{m}{}_{u}}{2}{\left(\frac{d}{dx}n_{u}+J_{u}\right)}^{2}-\frac{k_{mu}}{2}J_{u}^{2}+\frac{k_{t}}{2}{\left(n_{u}-\frac{d}{dx}H_{u}\right)}^{2}\\ +k_{cu}\left(n_{u}-\frac{d}{dx}H_{u}\right)\frac{d^{2}}{dx^{2}}n_{u}+k_{gru}{\left(\frac{d^{2}}{dx^{2}}n_{u}\right)}^{2}\\ +\frac{K_{a}}{2}{\left(-\frac{h_{u}{}^{2}\frac{d}{dx}n_{u}+2H_{u}-2M-2h_{u}}{2h_{u}}-\alpha_{0u}\right)}^{2}-\frac{K_{a}}{2}\alpha_{0u}^{2}\\ -\frac{B_{u}}{2h_{u}}\left(n_{u}-\frac{d}{dx}H_{u}\right)\left(h_{u}^{2}\frac{d^{2}}{dx^{2}}n_{u}+2\frac{d}{dx}H_{u}-2\frac{d}{dx}M\right)\\ -\frac{k_{cu}}{4h_{u}^{2}}{\left(h_{u}^{2}\frac{d^{2}}{dx^{2}}n_{u}+2\frac{d}{dx}H_{u}-2\frac{d}{dx}M\right)}^{2}\\ -\frac{C_{u}}{2h_{u}}\left(h_{u}^{2}\frac{d^{2}}{dx^{2}}n_{u}+2\frac{d}{dx}H_{u}-2\frac{d}{dx}M\right)\frac{d^{2}}{dx^{2}}n_{u}+\frac{S}{2}{\left(\frac{d}{dx}H_{u}\right)}^{2}\\ +\frac{k_{m}{}_{l}}{2}{\left(\frac{d}{dx}n_{l}+J_{l}\right)}^{2}-\frac{k_{ml}}{2}J_{l}^{2}+\frac{k_{t}}{2}{\left(n_{l}+\frac{d}{dx}H_{l}\right)}^{2}\\ +k_{cl}\left(n_{l}+\frac{d}{dx}H_{l}\right)\frac{d^{2}}{dx^{2}}n_{l}+k_{grl}{\left(\frac{d^{2}}{dx^{2}}n_{l}\right)}^{2}\\ +\frac{K_{a}}{2}{\left(-\frac{h_{l}{}^{2}\frac{d}{dx}n_{l}-2H_{l}+2M-2h_{l}}{2h_{l}}-\alpha_{0l}\right)}^{2}-\frac{K_{a}}{2}\alpha_{0l}^{2}\\ -\frac{B_{l}}{2h_{l}}\left(n_{l}+\frac{d}{dx}H_{l}\right)\left(h_{l}^{2}\frac{d^{2}}{dx^{2}}n_{l}-2\frac{d}{dx}H_{l}+2\frac{d}{dx}M\right)\\ -\frac{k_{cl}}{4h_{l}^{2}}{\left(h_{l}^{2}\frac{d^{2}}{dx^{2}}n_{l}-2\frac{d}{dx}H_{l}+2\frac{d}{dx}M\right)}^{2}\\ -\frac{C_{l}}{2h_{l}}\left(h_{l}^{2}\frac{d^{2}}{dx^{2}}n_{l}-2\frac{d}{dx}H_{l}+2\frac{d}{dx}M\right)\frac{d^{2}}{dx^{2}}n_{l}+\frac{S}{2}{\left(\frac{d}{dx}H_{l}\right)}^{2}. \end{array}$$

Varying this energy functional, we obtain the system of five linear Euler-Lagrange equations for the functions $n_{s}\equiv n_{u}+n_{b}$, $H_{m}\equiv H_{u}-H_{b}$, $H_{s}\equiv H_{u}+H_{b}$, M and $n_{m}\equiv n_{u}-n_{b}$. These equations are too bulky to be presented here. To solve these equations, we substitute the values of the elastic parameters, corresponding to the upper and lower monolayers depending on the membrane region under consideration (the L_o_ or L_d_ phase), and obtain the general solutions in the form of a sum $\sum_{i}C_{i}\exp(\lambda_{i}x)$, where $C_{i}$ and $\lambda_{i}$ are arbitrary and known complex constants, respectively. After adjusting the solutions to the real space, we substitute them to the boundary conditions and obtain the values of unknown constants $C_{i}$.

Our system consists of different regions, which represent different types of membranes: L_o_ bilayer, L_d_ bilayer, L_d_/L_o_ interface, and the region directly beneath the amphipathic peptides. At the boundaries of these regions, we impose the boundary conditions corresponding to the continuity of the neutral surfaces of the monolayers and director fields; at infinity, the projection of the director field to the *x*-axis is assumed to be equal to zero. Amphipathic peptides partially embedded into the monolayer push lipid heads apart, exposing the peptide hydrophobic and hydrophilic parts to lipid tails and heads, respectively. Therefore, amphipathic peptides are considered as an empty region at the upper monolayer; the width of the region is taken equal to 1.3 nm, i.e., the diameter of the α-helix. This is equivalent to the elastic moduli of the upper monolayer being equal to zero at this region. We note that the effect of the peptides on the membrane can be considered in two different ways. In the first (so-called “uniform”) approach, the peptides are considered as modifiers of the elastic parameters of the membrane, such as the spontaneous curvature or bending rigidity. This approach is most relevant on a large scale when the lipid membrane with embedded peptides can be considered as a laterally uniform medium. Within the second (“single peptide”) approach, the elastic parameters are defined by the lipid matrix and the peptides are considered separately as objects that impose constraints in terms of boundary conditions on the membrane deformations. The latter approach transforms to the first one when a large approximately uniform system is considered. The effects considered in the current work arise in nanometer scales. They are determined by local membrane perturbations induced by each peptide and do not involve the collective effect of the peptides as a component in the lipid-peptide mixture. Therefore, we employ the “single peptide” approach according to which the elastic parameters of the monolayers are determined by the lipid matrix, and peptides impose boundary conditions on the membrane deformations. The boundary conditions for amphipathic peptides take into account the difference in the projections of the directors at the peptides’ boundaries and the rotational degree of freedom of the peptides along the axis of the α-helix [36]: $\left|n_{2}-n_{1}\right|=\frac{\delta \Delta L}{\sqrt{{\left(\Delta L/2\right)}^{2}+{\left(h_{d}/2\right)}^{2}}}+\frac{\left(1-\delta \right)\Delta L}{\sqrt{{\left(\Delta L/2\right)}^{2}+{\left(h_{s}/2\right)}^{2}}}$ and $H_{u}(X_{0}+(\Delta L/2))-H_{u}(X_{0}-(\Delta L/2))=\Delta L\;(n_{1x}+n_{2x})/2$, where ΔL is the diameter of the α-helix (1.3 nm); δ is the fraction of the peptide diameter occupying the L_o_ monolayer; $h_{d}$ and $h_{s}$ are the hydrophobic thicknesses of the monolayers of the domain and surrounding membrane, respectively; $H_{u}$ is the shape of the neutral surface of the monolayer adjacent to the peptide; $X_{0}$ is the *x*-coordinate of the peptide’s center; $n_{1x}$ and $n_{2x}$ are the director projections onto the *x*-axis at the left and right boundary of the peptide, respectively. In the given expression for the director jump at the peptides’ boundaries, the directors are assumed to be directed towards the center of mass of the monolayer region occupied by the peptides if they are located in a homogeneous monolayer.

Besides, we take into account that L_o_ and L_d_ monolayers, located one above the other, laterally repel each other due to energetically unfavorable configurations of membrane thermal undulations [58,59]. Quantitatively, the corresponding energy density was determined experimentally [60] and estimated theoretically [58] as 0.016 *k*_B_*T*/nm^2^. We explicitly add this energy penalty to the total elastic energy of the L_d_/L_o_ interface.

## 3. Results

### 3.1. Structure of the L_o_/L_d_ Interface

**Figure 1:**
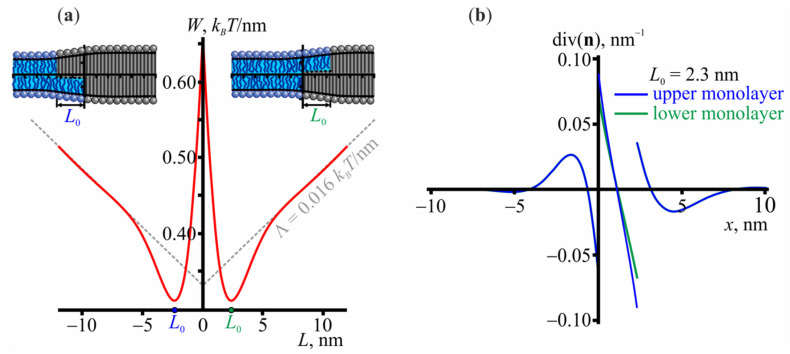
The structure of the L_o_/L_d_ interface. (**a**) The dependence of the elastic energy on the relative shift *L* between the boundaries of ordered monolayer domains located in opposing membrane leaflets. The membrane shapes are shown in the insets for the cases of |*L|* ≈ 2.3 nm, which correspond to the energy minimum. (**b**) The lateral distribution of the effective curvature, i.e., the divergence of the director, at the domain boundary. Blue and green colors correspond to the upper and lower monolayer, respectively.

Let us first consider the structure of the L_o_/L_d_ interface. Figure 1a shows the dependence of the elastic energy on the relative lateral shift *L* between the boundaries of ordered monolayer domains located in opposing membrane leaflets. *L* can be either positive or negative, which corresponds to the positive and negative coordinate of the boundary of the upper L_o_ domain, respectively. Due to the symmetry of the system, the energy is an even function of *L*. The energy reaches its minimum at |*L*| ≈ 2.3 nm and then monotonically increases. The monotonic increase primarily corresponds to the term Λ = 0.016 × *L k*_B_*T*/nm arising from the energy contribution of membrane undulations at large *L*; the elastic energy would be constant otherwise [58]. The membrane shape in the configurations corresponding to the minimum energy is shown in the insets of Figure 1a. Figure 1b shows the lateral distribution of the effective curvature, i.e., the director divergence, at the domain boundary and demonstrates that a non-zero curvature arises in a narrow region in the interface vicinity; the curvature rapidly decays far from the domain boundary.

### 3.2. Interaction of Amphipathic Peptides with the Domain Boundary

**Figure 2:**
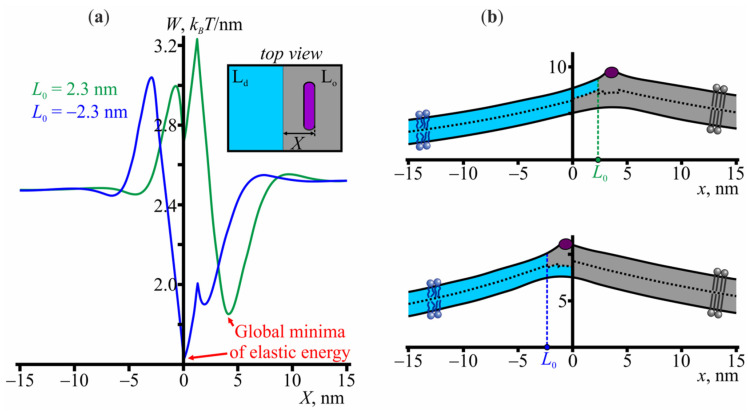
The interaction of amphipathic peptides with the L_o_ domain boundary. (**a**) The elastic energy profile as a function of the coordinate *X* of the peptide’s right boundary. The relative shift of the boundaries of the monolayers of the L_o_ domain is fixed at *L* = 2.3 nm (green curve) or *L* = −2.3 nm (blue curve), i.e., at the minimum of the energy profile shown in Figure 1a. (**b**) The membrane shape in configurations corresponding to the global energy minima at the panel (**a**); top—*L* = 2.3 nm, bottom—*L* = −2.3 nm.

Now, we add one amphipathic peptide to the system. We firstly fix the absolute value of the relative shift of the monolayer domain boundaries at |*L*| = 2.3 nm, which corresponds to the energy minimum in Figure 1a, and obtain the elastic energy for various positions of the amphipathic peptide. The results are presented in Figure 2a. As expected, the elastic energy is constant if the peptide is far from the boundary. However, as the peptide approaches the boundary, the energy deviates from the constant and reaches its global minimum when the peptide is located in the vicinity of the boundary. The energy is minimal at the coordinate of the peptide’s right boundary equal to *X*_0_ = 4.2 nm in the case of positive *L*, and *X*_0_ = 0 in the case of negative *L* (Figure 2b). The corresponding energy barriers, necessary for the peptide to escape from the energy well imposed by the domain boundaries, are 0.7 and 0.9 *k*_B_*T*/nm for the positive and negative *L*, respectively. It seems reasonable to propose that these configurations also correspond to the global minimum of the system, but if we allow the relative shift *L* to vary, thereby relaxing the elastic energy, we find that the global minimum configuration is slightly different. In the case of positive *L*, the global minimum appears at *L* = 0.9 nm and the coordinate of the peptide’s right boundary equal to 2.7 nm. In the case of negative *L*, the optimal value of *L* shifts to −2.4 nm and the optimal coordinate of the peptide’s right boundary remains at *X* = 0. Nevertheless, the key point of the analysis of the peptide/domain interaction is that the peptide strongly favors the region at the L_o_/L_d_ phase interface.

### 3.3. Membrane-Mediated Interaction of Amphipathic Peptides in a Homogeneous Membrane

**Figure 3:**
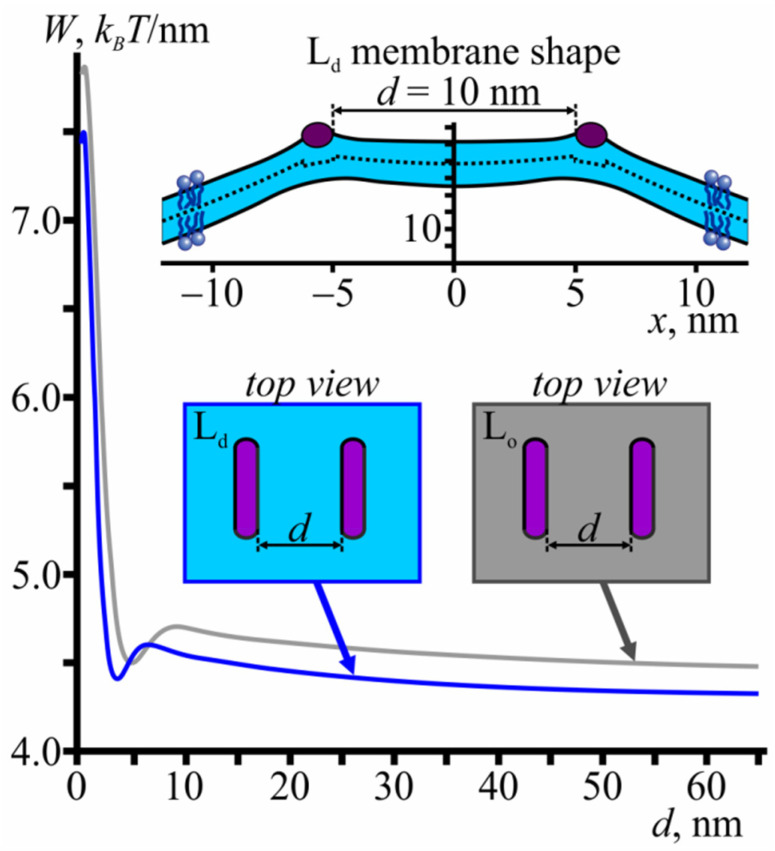
The membrane-mediated interaction of amphipathic peptides in a homogeneous membrane. Elastic energy profile as a function of the distance between the peptides in L_o_ (grey curve) and L_d_ (blue curve) membranes. The inset at the top of the figure shows the L_d_ membrane shape at a distance *d* = 10 nm between the peptides.

If amphipathic peptides are far from each other, the membrane deformations, induced by them, are independent. However, as the distance between the peptides decreases, the deformations overlap, leading to the membrane-mediated interaction between the peptides. Previously, it was shown that this interaction can be well described by a one-dimensional approach [44]. Figure 3 shows the elastic energy profiles as a function of the distance between the peptides in the L_o_ and L_d_ membranes.

Although there is a small local energy well at a distance *d* ≈ 5 nm, peptides mainly experience repulsion as they approach each other. The energy barrier that should be overcome to bring the peptides into contact with each other equals 3.2 *k*_B_*T*/nm and 3.4 *k*_B_*T*/nm for the L_d_ and L_o_ membranes, respectively (Figure 3).

### 3.4. Membrane-Mediated Interaction of L_o_ Domains

If two L_o_ domains are located far from each other, the elastic deformations at their boundaries are independent. At a small distance, however, the deformations overlap, which leads to the membrane-mediated interaction between the domains [23,46]. In general, to bring the boundaries of lipid domains into contact with each other, it is necessary to overcome some energy barrier; hereafter, we refer to this barrier as a fusion energy barrier. Now, we consider how the presence of amphipathic peptides, adsorbed into the membrane, influences the height of the fusion barrier. Given that: (i) amphipathic peptides prefer to occupy the boundary of the L_o_ domains; (ii) amphipathic peptides experience membrane-mediated repulsion as they approach one another, we expect that the domain-domain fusion barrier should increase when amphipathic peptides are added to the system. Before doing any exact calculations, we can already estimate the magnitude of this increase. Suppose that the number of amphipathic peptides adsorbed to the membrane is enough to completely occupy the boundaries of the L_o_ domains. As the distance between two L_o_ domains with amphipathic peptides at their boundaries becomes smaller, amphipathic peptides start to repel each other (see Figure 3). To avoid this energy rise, amphipathic peptides can increase the distance from each other but to do this they have to overcome the energy wells ~0.7−0.9 *k*_B_*T*/nm deep to escape from the boundaries of the domains (shown in Figure 2a); this depth, then, provides the required estimate of the increase of the fusion barrier height.

**Figure 4:**
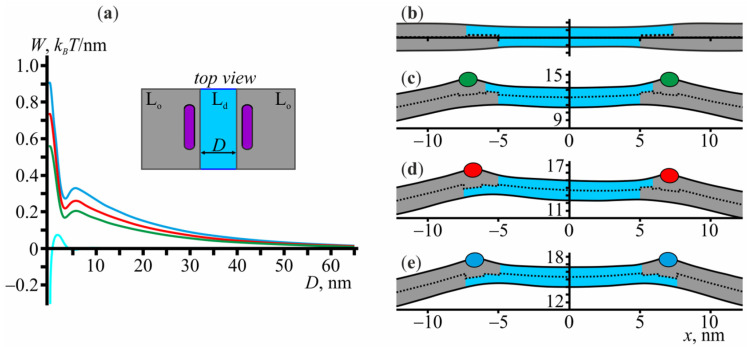
The effect of amphipathic peptides on the interaction of L_o_ domain boundaries. (**a**) The dependence of elastic energy of the membrane on the distance *D* between L_o_ domain boundaries in the absence (cyan curve) and presence (green, red, blue curves) of amphipathic peptides. (**b**–**e**) The calculated shapes of the membrane for a distance *D* = 10 nm between domain boundaries in the absence of the peptides (**b**) and the presence of the peptides at different configurations of the boundaries (**c**–**e**). In panels (**c**–**e**), the color of the peptide (shown as green (**c**), red (**d**), or blue (**e**) ellipses) corresponds to green, red and blue curves in panel (**a**), respectively.

Each bilayer L_o_ domain consists of two monolayer L_o_ domains, the boundaries of which can be relatively shifted. Thus, at a fixed minimum distance between the boundaries of two bilayer L_o_ domains, the system has four parameters that can vary: two relative shifts of the boundaries of monolayer L_o_ domains and two coordinates of the peptides, adsorbed at the boundary of each bilayer L_o_ domain. Varying the minimal distance between the domains and optimizing the elastic energy with respect to these four parameters, we can find the optimal trajectory of the domain-domain fusion and obtain the exact shape and height of the fusion energy barrier. In Figure 4a, we plot the elastic energy profile as a function of the distance *D* between the domains, where the energy is counted from that at the infinite distance (*D* → ∞). Here, the distance is defined as the minimum lateral distance between the monolayers of the L_o_ phase belonging to the different bilayer L_o_ domains (see the inset of Figure 4a). Firstly, we consider the interaction of the domains without amphipathic peptides (cyan curve in Figure 4a). At a given distance, the energy does not depend on the signs of relative shifts *L* of the L_o_ monolayer domains. The domains start to affect each other at a distance of about 5 nm and, to come into contact with each other, they have to overcome the energy barrier of 0.076 *k*_B_*T*/nm. The shape of the membrane at *D* = 10 nm is shown in Figure 4b.

Let us now consider the membrane-mediated interaction of two domains with amphipathic peptides adsorbed at their boundaries. Three cases are possible: the monolayers of the L_o_ phase are larger in the upper leaflet (Figure 4c), the sizes of the upper and lower L_o_ monolayers are different in both domains (Figure 4d), and the monolayers of the L_o_ phase are larger in the lower leaflet (Figure 4e). At *D* → ∞, the elastic energy of the membrane in all these configurations is about 3.3 *k*_B_*T*/nm. We note that along the optimum trajectory as *D* decreases, the configurations shown in Figure 4c,e always remain symmetric, i.e., the relative shifts *L* in both bilayer L_o_ domains remain equal to each other, and the peptides occupy the same position with respect to the L_o_ domain boundary. Among the three possible configurations of the boundary (Figure 4c–e), the smallest fusion energy barrier (~0.56 *k*_B_*T*/nm) corresponds to the configuration of Figure 4c, the largest one (~0.9 *k*_B_*T*/nm)—to the configuration of Figure 4e, the intermediate value (~0.73 *k*_B_*T*/nm)—to the configuration shown in Figure 4d. These values are approximately 7, 12, and 10 times larger than the fusion energy barrier of 0.076 *k*_B_*T*/nm for the fusion of the domains with no peptides at their boundaries. Overall, the optimal trajectory of the domain-domain fusion in the presence of amphipathic peptides corresponds to the green curve of Figure 4a with the configuration of Figure 4c, the L_o_ monolayers of which are larger in the lower leaflet. In plasma membranes of eukaryotic cells, however, unsaturation per lipid is higher in the cytoplasmic leaflet than in the exoplasmic one [1]. This implies that in living cells the exoplasmic monolayer of L_o_ domains is likely to be larger than the cytoplasmic one. As amphipathic peptides usually adsorb on the exoplasmic leaflet, among the three configurations of the domain boundary shown in Figure 4c–e, that of Figure 4e is the most relevant biologically. This configuration corresponds to the largest increase of the fusion energy barrier as compared to the case of the peptide-free membrane.

As the distance *D* between the domains decreases, in all possible configurations of the L_o_ domains (Figure 4c–e), it is energetically favorable for the peptides to stay in the vicinity of the boundaries of the L_o_ domains. However, the optimal relative shifts of L_o_ monolayer domains boundaries gradually increase from 0.9 nm to 1.9 nm in the case shown in Figure 4c, from 2.4 nm to 3.4 nm for the left domain and from 0.88 nm to 1.4 nm for the right domain in the case shown in Figure 4d, and from 2.4 nm to 3.5 nm in the case shown in Figure 4e. This implies that at a close distance between the domains, the elastic energy relaxes predominantly by tuning the relative shifts of the L_o_ monolayer domain boundaries. This shift gradually increases, which allows the peptides to keep a larger distance from each other and, at the same time, still occupy the optimal positions with respect to the boundaries, which correspond to the local minima of the elastic energy (Figure 2a).

## 4. Discussion

In this work, we have considered the membrane-mediated interaction of L_o_ domains in the presence of amphipathic peptides embedded into the membrane. Amphipathic peptides generate the local curvature and therefore tend to concentrate at the boundaries of L_o_ domains, where it is possible to minimize the overall curvature stress of the system. Thus, the pairwise repulsion between the peptides leads to the increase in the energy barrier of the L_o_ domains fusion. The fusion energy barrier is 0.076 *k*_B_*T*/nm in the system without amphipathic peptides. The barrier increases up to 0.56 *k*_B_*T*/nm, 0.73 *k*_B_*T*/nm, or 0.9 *k*_B_*T*/nm, depending on the configuration of the boundaries of L_o_ domains. To get the absolute values of the energy barriers, it is necessary to multiply the obtained values by the effective interaction length of two L_o_ domains, which can be estimated based on the Derjaguin approximation as $2\sqrt{2\lambda R}$ [61], where λ is the characteristic decay length of the elastic deformations and R is the domain radius. In our case, λ≈ 1 nm and thus for the L_o_ domain of radius R= 5 nm, for example, we get 0.5 *k*_B_*T* for the energy barrier of the domain-domain fusion without peptides, and 3.5 *k*_B_*T*, 4.6 *k*_B_*T*, 5.7 *k*_B_*T* with peptides in the different configurations of the boundaries of L_o_ domains as shown in Figure 4c–e. As outer monolayers of cell membranes are enriched in saturated lipids [1], lipid domains should be larger in the outer leaflet as compared to the cytoplasmic one. Thus, among the considered configurations, that of Figure 4e is the most relevant physiologically; for this configuration, the fusion energy barrier is 0.9 *k*_B_*T*/nm.

Amphipathic peptides are considered promising antibacterial, antiviral, and anticancer therapeutic agents [30,31,32,33,34,35]. The positive charge of these molecules allows them to selectively bind to the negatively charged membranes of bacteria or cancer cells, which are then destroyed via the formation of pores. However, these peptides can also bind to electrically neutral outer leaflets of plasma membranes of non-target eukaryotic cells via hydrophobic interactions. That is why much attention is devoted to the hemolytic activity of antimicrobial peptides. In this work, we considered another possible adverse side effect of antimicrobial peptides, whereby the lateral organization of the membranes is involved. The lipid composition of cell membranes is heterogeneous, which is responsible for the liquid-ordered/liquid-disordered phase separation [2,3,4,5,6,7,8] with nanoscopic L_o_ domains floating in the L_d_ membrane [3,5,6]. Several processes such as signal transduction [16,24,25], polarized sorting [26], and creation of immunological synapses [27] are proposed to be regulated by a so-called raft clustering, whereby ordered nanodomains fuse to create one larger domain. It is known from experiments that this clustering is involved in neuronal polarity determination [28] and assembly of integrin signaling complexes [29]. Therefore, misregulation of L_o_ domains fusion events might lead to undesirable adverse side effects. Indirectly, these effects are manifested in Smith-Lemli-Opitz syndrome [21], whereby improper cholesterol biosynthesis and its partial substitution by its metabolic precursor significantly hinders the fusion of L_o_ domains [23]. Results of the present work show that amphipathic peptides should also cause this impediment to the domain-domain fusion. After adsorption to the membrane with pre-existing phase separation, peptides are predicted to occupy the boundaries of L_o_ domains [36]. We previously showed that this accumulation of the peptides at the boundaries of L_o_ domains should reduce the probability of the formation of pores in the membrane [62]. At the same time, the pairwise repulsion between the peptides [43] should give rise to the corresponding membrane-mediated repulsion between L_o_ domains. Actually, as we have shown, the energy barrier of the fusion of two domains with peptides adsorbed at their boundaries is 12 times larger than the corresponding barrier for domains without peptides. Given the large involvement of raft clustering in various cell processes, we suggest that the increase in the energy barrier of the fusion of L_o_ domains might cause adverse side effects, apart from hemolytic activity, in clinical applications of antimicrobial peptides.

While in model lipid membranes, both microscopic and nanoscopic domains are observed [63,64], in cell membranes domains are nanoscopic [3,5,6]. It is proposed that the main determinant of the domain size distribution is the line tension at their boundary [65]. It is entropically favorable for the system to be dispersed into numerous small domains. At the same time, at high line tensions, it is energetically more advantageous to form a single macroscopic domain. The experimentally determined value of the critical line tension driving nanoscopic to macroscopic domain transition is 0.3 pN [63]. From our results, it follows that, although amphipathic peptides increase the line tension (compare minimum energy values in Figure 1 and Figure 2), the ensemble of nanoscopic domains is nonetheless stabilized due to the increased domain fusion energy barrier. Cell membranes contain a lot of components, other than peptides, which can also induce local curvature. We previously showed that lipids with nonzero spontaneous curvature tend to accumulate at the boundary of L_o_ domains [45]. Experimentally it was shown that one of such lipids, ganglioside GM1, stabilizes the ensemble of nanoscopic domains [66,67]. The question, whether in cells the curvature inducing membrane components might also contribute to the stabilization of the size of L_o_ nanodomains, requires additional exploration.
